# Association between animal welfare indicators and microbiological quality of beef carcasses, including *Salmonella* spp., from a slaughterhouse in Ecuador

**DOI:** 10.14202/vetworld.2021.918-925

**Published:** 2021-04-16

**Authors:** María Cevallos-Almeida, Ana Burgos-Mayorga, Carlos A. Gómez, José Luis Lema-Hurtado, Leydi Lema, Iveth Calvache, Christian Jaramillo, Isabel Collaguazo Ruilova, Evelyn Pamela Martínez, Pamela Estupiñán

**Affiliations:** 1 Laboratorio de Bacteriología y Micología, Facultad de Medicina Veterinaria y Zootecnia, Universidad Central del Ecuador, Quito, Ecuador; 2 Facultad de Medicina Veterinaria y Zootecnia, Universidad Central del Ecuador, Quito, Ecuador

**Keywords:** animal welfare, beef carcasses, *Escherichia coli*, *Salmonella* spp, total aerobic bacteria

## Abstract

**Background and Aim:**

Pre-slaughter management and slaughter operations are considered critical factors for animal welfare and meat quality. Previous studies have found poor animal welfare management at municipal slaughterhouses in Ecuador, and little is known about how this affects the microbiological quality of the meat. Therefore, the aim of the study was to analyze the association of the microbiological quality of beef carcasses and animal welfare indicators in a municipal slaughterhouse in Ecuador.

**Materials and Methods:**

Data for 6 months were collected from a municipal slaughterhouse in Ecuador. Five trained researchers were strategically located along the slaughter process. A total of 351 animals were observed with regard to welfare indicators, and their carcasses were sampled to evaluate microbiological quality. Antemortem (slipping, falling, and vocalization) and postmortem animal welfare indicators (bleed interval, pH, temperature, and bruises) were measured. To determine the total aerobic bacteria (TAB) and *Escherichia coli* counts and the presence of *Salmonella* spp., we collected samples by swabbing four different points of each carcass. The association between microbiological quality and animal welfare indicators was studied using univariate and multivariate logistic regressions.

**Results:**

The mean TAB count was 5.3 log CFU/cm^2^, and the mean total count of *E. coli* was 2.4 log CFU/cm^2^. *Salmonella* spp. were isolated in 3.1% of the carcasses. An electric goad was used in all animals, 19.1% slipped at least once, and 19.9% vocalized. The mean pH of the carcasses was 7.2, and 79.2% of carcasses had bruises. Multivariate analysis showed that *Salmonella* spp. and the TAB count were associated with pH and the number of bruises (p = 0.01 in both cases).

**Conclusion:**

Although there was non-significant association between the majority of animal welfare indicators and microbiological quality, the poor management affecting animal welfare and carcass hygiene are worrisome.

## Introduction

Beef production is an important global economic activity, especially in developing countries. In Latin America and the Caribbean, livestock is considered the basic protein source for food security, and the consumption of animal-sourced proteins is considered the most important strategy to bolster the health of local populations [1]. In Ecuador, the cattle population is approximately 4.13 million heads, located mainly in rural areas [2], and 84% of rural households own livestock, with an average of 2.8 heads per household [1]. In 2016, Ecuadorians were estimated to have consumed 182,000 tons/year of beef, with 17.6 kg of beef per person per year [3].

Pre-slaughter and slaughter operations are considered the most important critical factors for meat quality and food security [4]. Pre-slaughter factors, such as failures in handling practices during loading and unloading and during transport, unskilled drivers, long-distance travel, high density during transport, and inadequate lairage facilities at the slaughterhouse (e.g., privation of water), can induce stress in animals and decrease animal welfare [5-7]. Stress, in turn, can induce metabolic and hormonal alterations, leading to changes in the color, pH, and water-holding capacity of the meat [8]. In addition, bad slaughter management during unclean (e.g., stunning and bleeding) and clean (e.g., evisceration and carcass splitting) operations can affect the carcass quality due to cross-contamination [9]. Post-slaughter factors, such as temperature, humidity, and storage time, also affect the final microbiological quality of the meat [10].

In many countries, the identification of microorganisms such as *Salmonella* spp., the total aerobic bacteria (TAB) count, and the *Escherichia coli* count are used as criteria for assessing good slaughter hygiene in beef [11]. Therefore, microbiological data are essential to implement meat-quality-monitoring programs to prevent the transmission of common foodborne diseases [12]. In Ecuador, most slaughterhouses are public and operated by municipalities providing slaughter service at subsidized rates to wholesalers. Service quality is low with regard to animal welfare and humane handling practices [13]. The demand for high-quality beef is increasing, and consumers are willing to pay more for products obtained with better animal welfare and sanitary control [13]. However, previous studies have found poor animal welfare management at municipal slaughterhouses in Ecuador [14-17], and little is known about how this affects the microbiological quality of the meat.

Therefore, this study determined the association between animal welfare indicators and the microbiological quality of beef carcasses from a municipal slaughterhouse in Ecuador.

## Materials and Methods

### Ethical approval

Ethical approval was not necessary for this study since it merely focused on observing the animal welfare management performed at the slaughterhouse, and no questionnaire survey was performed. However, the skin painted used on selected animals was done without any harm, and the microbiological samples were collected according to standardized methods.

### Study design, period, location, and sampling

This cross-sectional study was conducted from November 2018 to April 2019 in a slaughterhouse in Ecuador. The slaughterhouse provides slaughter service to wholesalers coming from the coast, highlands, and Amazon regions of Ecuador. On an average, 500 animals are slaughtered every day. On the basis of this number, we calculated a desired total sample size of 351 animals at a 95% confidence interval (CI). To ensure correct observation of animal welfare indicators and considering the time constraints for bacterial isolation, we randomly selected an average of 25 animals from pens 1 day/week (only Mondays).

Five researchers were strategically located along the slaughter process, from pens to the final point in the slaughter line commercial area. For sampling, the researchers talked to each other using walkie-talkies to follow individual carcasses. Each selected animal was assigned a number, which was drawn on the animal’s skin on both the head and the flank with blue paint. Once the skin was removed at the first point of the slaughter line, the carcass was identified with a livestock tag ring with the same number. The tag ring was attached around the animal’s Achilles tendon, which was removed after sampling. This procedure has been used in previous studies in the same slaughterhouse and was found to be useful to follow animals and carcasses. Workers at the slaughterhouse saw these marks during every trial, but they kept doing their job as usual [15,17].

### Assessment of animal welfare

We evaluated antemortem and postmortem animal welfare indicators using the recommendations of the North American Meat Institute (NAMI) and the World Organization for Animal Health [18,19]. Antemortem indicators were observed directly and included the number of times an animal slipped and fell, the number of vocalizations, and the use of an electric goad (yes/no). Slipping, falling, and vocalizations were counted during animal handling from the holding pen to the stunning box. Falling was counted as loss of contact of all four legs with the floor or the belly of the animal touching the floor. Slipping was counted as any loss of footing. Vocalizations were measured only when they occurred due to the electric goad, hits by handlers, or other abusive actions, such as holding the animals by the horns. The use of an electric goad was counted when electric discharge was effectively done [18].

Postmortem indicators were measured after stunning and included the number of shots needed for stunning, the stunning-to-bleed interval, pH, temperature, and the number of bruises. The stunning-to-bleed interval was defined as the time from effectively stunning (i.e., unconsciousness) the animal to cut the blood vessels in the neck and upper chest and was measured in seconds. To evaluate the return to consciousness, we observed the animal’s eye reflexes (e.g., corneal reflexes and mydriasis), reaction to pain, rhythmic breathing, and vocalizations [18]. Any tissue lesion with a decoloring zone due to the rupture of blood capillaries was considered a bruise [20]. Bruises were counted in four regions: (1) The front, from the neck to the front leg; (2) the back, the muscles around the spine from the shoulders to the lumbar region; (3) the ribs, the muscles in the flanks; and (4) the rump and rear legs. The pH and temperature were measured 45 min after stunning, using a portable potentiometer (Hannah Instruments,USA) with a penetration ­electrode in the *Longissimus dorsi* muscle. The latter procedure was in accordance with the Ecuadorian standard INEN-ISO 2917:2013 [21].

### Isolation and bacterial identification

Microbiological analyses were performed in the Laboratory of Bacteriology at the Faculty of Veterinary Medicine and Zootechnics, Central University of Ecuador. Carcasses marked with livestock tag rings were sampled for microbiological quality at the end of the slaughter line after washing.

Samples were collected using a nondestructive swab method with a sponge soaked in buffered peptone water [22]. Samples were taken at four points identified as highly contaminated areas: Hip, skirt, chest, and neck [23]. The sampling area was 100 cm^2^, defined by a sterile plastic frame. Subsequently, the sponge was placed in a Ziploc bag and kept at 4°C [22]. Next, the samples were diluted (1:10) in buffered peptone water (Difco, USA) and then serially diluted in 0.45% saline solution until a dilution of 10^–3^ was obtained. Then, 100 mL of dilutions 10^–2^ and 10^–3^ was plated on Chromocult Agar (Merck, USA) for the *E. coli* count and Plate Count Agar (BD-Difco, USA) for the TAB count (CFU/cm^2^); the estimation was done according to the ISO 7218 standard [24].

*Salmonella* spp. isolation was done according to the ISO 6579 standard [25]. Briefly, three drops of the initial suspension were dispensed on Modified Semi-Solid Rappaport Vassiliadis (BD-Difco) and incubated at 42°C for 24-48 h. Presumptive colonies were observed and classified according to their growth and migration (CM), growth without migration (CNM), and non-growth (NC). A loopful from the edge of the migration zone was streaked into a xylose lysine deoxycholate agar plate (BD-Difco) and incubated at 37°C for 24 h. For confirmation, two presumptive *Salmonella* spp. colonies were tested using Triple Sugar Iron agar (BD-Difco), Lysine Iron agar (BD-Difco), Urea agar (BD-Difco), and Sulfur Indole Motility medium (BD-Difco) [26].

### Statistical analysis

Statistical analyses were performed using R Cran 3.6.1 and R Studio version 1.1.442 (R Foundation for Statistical Computing, Vienna, Austria, URL https://www.R-project.org/). TAB and *E. coli* counts were expressed as log CFU cm^2^ and compared by reference to mean values. *Salmonella* spp. were expressed as their absence and presence. Reference values for animal welfare indicators were based on NAMI and Food and Agriculture Organization guidelines and similar studies [18,27-29]. Reference values for microbiological quality parameters were based on European Union guidelines and the Food Security Criteria from Argentina [30,31].

Visual inspections of histograms showed a clear binomial distribution of TAB and *E. coli* counts. Therefore, these variables were categorized for further analysis as below and above reference values. The above category for the TAB count was applied for counts more than 5.0 log CFU/cm^2^ and for *E. coli* counts more than 2.5 log CFU/cm^2^.

To explore the association of microbiological parameters with welfare indicators, logistic regression models were applied to calculate crude and adjusted odd ratios (ORs) and their 95% CIs. Univariate analysis was used to identify potential explanatory variables to be included in multivariate analysis. Models were fitted separately with the TAB, *E. coli*, and *Salmonella* spp. as binary outcome variables. The following explanatory variables were considered: Falling (yes/no, counts), slipping (yes/no, counts), vocalizations (yes/no, counts), bruises (yes/no, counts), temperature, and pH.

Multivariate models were built using forward stepwise selection, and the model with the lowest Akaike information criterion (AIC) was considered the best. The AIC has been widely used to evaluate the goodness of fit [32]. Variables that were not statistically significant and did not improve the AIC were excluded from the models.

## Results

### Microbiological quality and animal welfare parameters

Table-1 shows the parameters related to animal welfare and the microbiological quality of the carcasses. Aerobic bacteria were isolated in 100% of the carcasses, and *E. coli* and *Salmonella* spp. were isolated in 34.5% (121/351) and 3.1% (11/351) of the carcasses, respectively. The TAB count was 4.1-6.43 log CFU/cm^2^, and 81.8% (287/351) of the samples were above the reference limit of 5 log CFU/cm^2^. The *E. coli* count was 0-5.4 log CFU/cm^2^, and 65.5% (230/351) of the samples were above the reference limit of 2.5 log CFU/cm^2^. With regard to antemortem indicators, 7.7% (25/351) of the animals fell and 19.08% (67/351) slipped on the way to the stunning box. An electric goad was used in all observed animals, with a mean of 5.2±4.2. In addition, 19.9% (70/351) of the animals vocalized due to human handling, with a mean of 0.7±3.4 vocalizations per animal. The mean stunning-to-bleed interval was 135.8±107.2 s.

**Table-1:** Summary data of parameters regarding microbiological quality in beef carcasses and animal welfare indicators.

Parameters	n (%)	Mean (SD)	Range	Reference values^a^	Above reference

					n (%)
Microbiological quality					
TAB (log UFC/cm^2^)	351 (100.0)	5.32 (0.39)	4.1-6.4	3.5-5.0	287 (81.8)
*E. coli* (log UFC/cm^2^)	121 (34.4)	2.4 (1.8)	0.0-5.4	0.7-2.5	230 (65.5)
*Salmonella* spp.	11 (3.1)	-	-	Absence	11 (3.1)
Animal welfare indicators					
Antemortem					
Falling	25 (7.1)	0.1 (0.3)	0.0-1.0	<1%	25 (7.1)
Slipping	67 (19.1)	0.27 (0.7)	0.0-5.0	<3%	67 (19.1)
Vocalizations	70 (19.9)	0.7 (3.4)	0.0-49.0	<3%	70 (19.9)
Electric prod usage	351 (100.0)	5.2 (4.2)	1.0-32.0	<25%	351 (100.0)
Postmortem					
Bruising	278 (79.2)	2.5 (2.2)	0.0-9.0	NA	NA
Front	24 (8.6)	-	-	-	-
Back	7 (2.5)	-	-	-	-
Ribs	28 (10.1)	-	-	-	-
Rump	219 (78.8)	-	-	-	-
Subcutaneous	181 (65.1)	-	-	-	-
Muscle	97 (34.9)	-	-	-	-
S/B interval (s)	-	135.8 (107.2)	31.0-1282.0	<60 s	300 (85.5)
pH_ 45_	-	7.2 (0.3)	5.8-8.0	<7	237 (67.5)
Temperature (°C)	-	29.9 (1.3)	25.0-34.1	<35°C	0 (0.0)

NA=Not applicable, S/B interval, stunning bleed interval measured in seconds; pH_ 45,_ pH measured 45 h after slaughter.

a Reference values for microbiological quality were based on a combination of regulations from the European Union and Argentina, and for animal welfare indicators were mainly based on the NAMI and FAO guidelines. Reference values for antemortem animals welfare indicators refer to the maximum percentage of animals over the total observed animals. There is no reference value for number of bruises. *E. coli=Escherichia coli*, TAB=Total aerobic bacteria

With regard to postmortem indicators, we found bruises in 79.2% (278/351) of the carcasses, with a mean of 2.5±2.2 per carcass. The majority of bruises were located on the rump (78.8%) and were subcutaneous (65.1%). The mean pH was 7.2, with 46.1% (166/351) of the carcasses having pH values of >7.3. The mean temperature in the sampled carcasses kept in the commercial area was 29.9°C (Table-1).

### Association between animal welfare indicators and microbiological quality of carcasses

Tables-2 and 3 show the ORs for high TAB and *E. coli* counts and the presence of *Salmonella* spp. Most of the antemortem and postmortem animal welfare indicators were not associated with high TAB and *E. coli* counts and the presence of *Salmonella* spp. in beef carcasses. Interestingly, univariate analysis revealed that the increase in pH significantly decreased the odds for high TAB counts (OR=0.34, 95% CI=0.13-–0.80, p=0.02) and the presence of *Salmonella* spp. (OR=0.08, 95% CI=0.02-0.43, p=0.003) (Table-2). This effect was still significant when adjusting for slipping and vocalizations for the TAB count (OR=0.28, 95% CI=0.11-0.70, p=0.01) and the number of bruises for *Salmonella* spp. (OR=0.10, 95% CI=0.02-0.51, p=0.01) (Table-3).

**Table-2:** Univariate odds ratio for the presence of Aerobic Bacteria, *E. coli* and *Salmonella* spp. based on animal welfare indicators.

Welfare indicators	Presence of aerobic bacteria			Presence of *E. coli*			Presence of *Salmonella* spp.		
		
	n (%)	OR (95% CI)	p-value	n (%)	OR (95% CI)	p-value	n (%)	OR (95% CI)	p-value
		
	Mean (SD)			Mean (SD)			Mean (SD)		
Antemortem									
Falling									
No	267 (81.9)	Ref.		212 (65.0)	Ref.		9 (2.8)	Ref.	
Yes	20 (80.0)	0.88 (0.31-3.14)	0.81	18 (72.0)	1.38 (0.58-3.65)	0.48	2 (8.0)	3.06 (0.45-12.78)	0.19
Slipping									
No	228 (80.3)	Ref.		187 (65.9)	Ref.		8 (2.8)	Ref.	
Yes	5 (88.6)	1.81 (0.80-4.63)	0.14	43 (64.2)	0.93 (0.54-1.64)	0.80	3 (4.5)	1.62 (0.35-5.77)	0.49
Counts	0.3 (0.7)	1.30 (0.85-2.31)	0.55	0.3 (0.7)	1.02 (0.75-1.43)	0.91	0.3 (0.7)	1.0 (0.30-1.87)	0.99
Vocalization									
No	232 (82.6)	Ref.		182 (64.8)	Ref.		10 (3.6)	Ref.	
Yes	55 (78.6)	0.74 (0.41-1.52)	0.44	48 (68.6)	1.19 (0.68-2.11)	0.55	1 (1.4)	0.39 (0.02-2.10)	0.38
Counts	0.7 (3.4)	0.96 (0.89-1.05)	0.19	0.7 (3.4)	0.97 (0.90-1.04)	0.39	0.7 (3.4)	1.09 (1.01-1.20)	0.02
*Electric prod* use	5.2 (4.2)	0.98 (0.93-1.05)	0.55	5.2 (4.2)	1.02 (0.97-1.08)	0.43	5.2 (4.2)	1.04 0.90-1.15)	0.51
Postmortem									
Bruises									
No	58 (79.5)	Ref.		45 (61.6)	Ref.		6 (8.2)	Ref.	
Yes	229 (82.3)	1.21 (0.59-2.38)	0.57	185 (66.6)	1.24 (0.72-2.10)	0.43	5 (1.8)	0.2 (0.06-0.70)	0.01
Counts	2.5 (2.2)	0.98 (0.86-1.11)	0.69	2.5 (2.2)	0.98 (0.89-1.09)	0.74	2.5 (2.2)	0.96 (0.70-1.26)	0.78
S/B interval (s)	135.8 (107.2)	1.00 (1.00-1.00)	0.49	135.8 (107.2)	1.00 (1.00-1.00)	0.06	135.8 (107.2)	1.0 (0.99-1.00)	0.46
pH	7.2 (0.33)	0.34 (0.13-0.80)	0.02	7.2 (0.33)	0.53 (0.26-1.05)	0.07	7.2 (0.33)	0.08 (0.02-0.43)	0.003
Temperature (°C)	29.9 (1.3)	0.95 (0.69-1.33)	0.78	29.9 (1.3)	1.1 (0.93-1.30)	0.27	29.9 (1.3)	1.17 (0.74-1.91)	0.52

OR=Odds ratio, Ref., reference category, S/B interval, stunning bleed interval measured in seconds. *E. coli=Escherichia coli*

**Table-3:** Multivariate odds ratio for the presence of aerobic bacteria (Model A) and *Salmonella* spp. (Model B) based on animal welfare indicators.

Variables	n/n (%)	OR	95% CI	SE	p-value

	mean (±SD)				
Model A: Presence of aerobic bacteria					
Slipping					
No	228/284 (80.3)	Ref.			
Yes	59/67 (88.1)	2.10	0.97-3.86	0.42	0.08
Vocalizations					
Counts	0.73 (3.4)	0.94	0.87-1.01	0.03	0.07
pH	7.17 (0.3)	0.28	0.11-0.70	0.47	0.01
Model B: Presence of *Salmonella* spp.					
Bruises					
0	6/73 (8.2)	Ref.			
1-4	2/214 (9.3)	0.11	0.02-0.52	0.84	0.01
5-9	3/64 (4.7)	0.55	0.11-2.30	0.75	0.43
pH	7.17 (0.33)	0.10	0.02-0.51	0.87	0.01

OR=Odds ratio, Ref., reference category. Model A and Model B included variables based on its significant and Akaike information criterion

In addition, the presence of *Salmonella* spp. was significantly lower with the presence of bruises (OR=0.20, 95% CI=0.06-0.70, p=0.01) and when the carcasses had one to four bruises (OR=0.11, 95% CI=0.02-0.52, p=0.01). However, the number of vocalizations was weakly associated with the presence of *Salmonella* spp. (OR=1.09, 95% CI=1.01-1.20, p=0.02), but this effect disappeared when included in the multivariate model (Table-3). We did not find a significant effect of animal welfare indicators on *E. coli* presence. However, the odds for having the *E. coli* count increased by ~20-30% when the animal fell and the carcasses had bruises (Table-2).

## Discussion

This study analyzed the microbiological quality of beef carcasses, including the TAB count, the *E. coli* count, and the presence of *Salmonella* spp., and its association with animal antemortem (vocalization, slipping, falling, and use of an electric prod) and postmortem (bruises, stunning/bleed interval, pH, and temperature) animal welfare indicators in a municipal slaughterhouse in Ecuador. The majority of animal welfare indicators were not associated with microbiological quality. However, there was evidently poor welfare management.

A higher pH reduced the risk of the presence of *Salmonella* spp. and a TAB count of >5.0 log CFU/cm^2^. Probably, this result is an effect of the measurement of pH 45 min after stunning, resulting in a mean of 7.1. According to NAMI guidelines, the ­measurement of postmortem indicators, such as pH, temperature, and bruises, should be done 24 h after slaughter [18]. However, the slaughterhouse in this study only provides slaughter service, and beef carcasses are dispatched to wholesalers immediately after slaughter. Therefore, postmortem indicators were measured just before dispatch. The pH, water activity, and temperature of carcasses are factors that affect microbial growth and survival. In fact, pH values of >9 or <4 can inhibit *Salmonella* spp. growth [31].

Our results showed that the risk of the presence of *Salmonella* spp. is low when the number of bruises is high, regardless of the fact that 79.2% of the carcasses had at least one bruise. The presence of bruises has been widely used as an indicator of animal welfare [33-35]. Its presence is associated with poor pre-slaughter handling factors related to transport, food and water privation, lairage, and stunning, which lead to physical distress and subsequent changes in meat quality [4]. For instance, food and water deprivation of 12-24 h results in a significantly higher muscle pH (up to 5.6, p<0.05) in poultry, and it increases the number of bruises in pigs [36,37]. Although these effects are hard to see in cattle after 12-24 h [38], it is possible that both the presence of bruises and a pH of 7 could lower the risk of the presence of *Salmonella* spp. as much as it did in our study (OR=0.10, p=0.01), in addition to TAB and *E. coli* counts. However, further studies are necessary to fully understand these associations by measuring pH and bruises after 24 h and including samples from bruises for microbiological analysis.

Surprisingly, we did not find a significant association between antemortem animal welfare indicators and microbiological quality, regardless of the evidently poor management, probably because the true effect of animal welfare indicators was not captured due to a high level of carcass contamination. Indeed, 81.8% of the carcasses had a TAB count higher than the reference value of 5 log CFU/cm^2^, and 65.5% of the carcasses had an *E. coli* count higher than 2.5 log CFU/cm^2^. Although not significant, slipping could double the risk of having a high TAB count. Slipping, falling, and vocalization have been related with the high use of an electric goad [34,39] and poor infrastructure [40,41]. In this study, 100% of the observed animals were prodded toward the stunning box using an electric goad, of which 7.1% fell, 19.1% slipped, and 19.9% vocalized. Of note, none of these antemortem indicators complied with NAMI guidelines [18], reflecting the serious shortcoming of animal welfare management at the slaughterhouse. These results should be studied further to increase awareness of pre-slaughter management, which certainly could affect meat quality traits such as color, pH, conductivity, shelf life, and water retention [8].

The mean TAB (5.3 log CFU/cm^2^) and *E. coli* (2.4 log CFU/cm^2^) counts found in this study were comparable with the previous studies. Two studies in Brazil reported a mean *E. coli* count of 2.11 log CFU/cm^2^ and 2.57 log CFU/cm^2^, respectively, in beef carcasses [12,42]. A study in Europe reported a mean *E. coli* count of 2.5 log CFU/cm^2^ in beef carcasses and a lower mean of 0.9 log CFU/cm^2^ in sheep carcasses [43]. We found high variability in the *E. coli* count, ranging from 0.0 to 5.4 log CFU/cm^2^, probably due to the geographical diversity of animals arriving at the slaughterhouse, their age, hygiene, and facility cleaning [42,44]. In contrast with our findings, TAB counts were higher than the counts reported from samples collected after bleeding cattle at a slaughter line in Brazil (4.51 log CFU/cm^2^) [12].

In addition, 3.1% of our samples were positive for *Salmonella* spp. compared to five slaughterhouses in the coastal region of Ecuador where this bacterium was not isolated from 70 samples [10]. However, this percentage was low compared to other studies. In three Brazilian slaughterhouses, 6.7% of the 90 samples collected at three different points in the slaughter line (stunning, washing, and cooling) were positive for *Salmonella* spp. [45]. Besides, a study in Mexico isolated *Salmonella* spp. from 18% of the carcasses, 39% of beef chunks, and 71% of ground beef samples, indicating that the *Salmonella* spp. prevalence tends to increase with meat processing [46]. The variability in the presence of *Salmonella* spp. can also be explained by the size of the establishment. One study found a much higher *Salmonella* spp. prevalence in beef carcasses in a small-scale (<500 employees) slaughterhouse compared to that of our study (58% vs. 3.2%), possibly due to the fact that samples in that study were taken pre-evisceration, before the washing process [47]. In our study, carcasses were sampled after washing, which may have lowered the load of *Salmonella* spp. [48].

Our study had a few strengths but also some limitations. This is the first study covering the association of the microbiological quality of beef carcasses and animal welfare indicators in a municipal slaughterhouse in Ecuador. Although we could not find a strong association between them, the measured indicators were useful to describe the hygiene and animal welfare management in the slaughterhouse, which only provides slaughter service. One limitation was the inability to measure postmortem animal welfare indicators 24 h after slaughter, which made it difficult to compare our results with other studies. Nevertheless, our findings showed worrisome poor welfare and hygiene management consistent with the previous studies performed in the same slaughterhouse [15,17]. A high bacteria count in the first point of the slaughter line suggests higher contamination by the end of the slaughter process [12,46,49]. Therefore, further studies should consider using different sampling points along the slaughter line. Another limitation was the lack of information regarding animal demographic factors, transportation, and handling during lairage, since these are not routinely collected. These factors can affect the microbiological quality of meat, as shown in other studies [50,51].

## Conclusion

The majority of animal welfare indicators were not associated with the microbiological quality of beef carcasses. Only pH and bruises are associated with a low risk of the presence of *Salmonella* spp. and the TAB count. More importantly, the municipal slaughterhouse in Ecuador has a worryingly low microbiological quality of beef carcasses and poor animal ­welfare management, indicating the necessity of further studies on these two aspects.

## Authors’ Contributions

AB, EPM, PE, and MC conceived and designed the study. CAG, MC, LL, and JLL did the microbiology analysis at the laboratory. IC, ICR, and CJ, measured animal welfare indicators at the slaughterhouse. EPM and AB analyzed the data. EPM, AB, MC, and PE interpreted the data, wrote, and revised the manuscript. All authors read and approved the final manuscript.
